# Repeated cobalt and chromium ion measurements in patients with large-diameter head metal-on-metal ReCap-M2A-Magnum total hip replacement

**DOI:** 10.1080/17453674.2019.1595469

**Published:** 2019-04-04

**Authors:** Heikki Mäntymäki, Petteri Lankinen, Tero Vahlberg, Aleksi Reito, Antti Eskelinen, Keijo Mäkelä

**Affiliations:** a Department of Orthopaedics, Tampere University Hospital and University of Turku;;; b Department of Orthopaedics and Traumatology, Turku University Hospital and University of Turku;;; c Department of Biostatistics, University of Turku;;; d Coxa Hospital for Joint Replacement and University of Tampere, Finland

## Abstract

Background and purpose — Whole blood (WB) cobalt (Co) and chromium (Cr) ion levels have a major role in the follow-up of metal-on-metal total hip replacement (MoM THR). We investigated, first, if there was a change in WB Co or Cr levels over repeated measurements in patients with ReCap-M2A-Magnum THR, and, second, determined how many patients had WB Co or Cr levels that exceeded the safe upper limits (SUL) in the repeated whole blood metal ion assessment.

Patients and methods — A Recap-M2A-Magnum THR was used in 1,329 operations (1,188 patients) at our institution between 2005 and 2012. We identified all patients (n = 319) with unilateral ReCap-M2A-Magnum implants who had undergone at least 2 repeated metal ion measurements with the first blood sample taken mean 5.5 years (1.8–9.3) after surgery and the second taken mean 2 years (0.5–3) after the first.

Results — The median WB Co and Cr ion levels decreased in repeated measurements from 1.40 (0.40–63) ppb to 1.10 (0.20–68) ppb and from 1.60 (0.60–13.0) ppb to 1.10 (0.30–19.0) ppb, respectively. 7% of the Co ion values exceeded SUL at the initial measurement, and 7% at the control measurement. The proportion of Cr ion values exceeding the safe upper limit (SUL) decreased during the measurement interval from 5% to 4%.

Interpretation — Repeated metal ion measurements in unilateral ReCap-M2A-Magnum patients in a mean 2-year time interval did not show any increase. Long-term ion levels are, however, not yet known.

Metal debris-related local reaction (adverse reaction to metal debris, ARMD) is a well-known complication of large-diameter head metal-on-metal total hip replacement (MoM THR). More than 1 million MoM THRs were performed before widespread concerns about ARMD were raised (Lombardi et al. 2012). The Australian Orthopaedic Association’s National Joint Replacement Registry (AOANJRR) was the first to report early failures of MoM THR (AOANJRR 2008). Despite the AOANJRR’s report, it took approximately 4 years for the European orthopedic community to adequately react to the issue (MHRA 2012). In Finland, the Finnish Arthroplasty Society recommended, in 2012, not to continue implantation of MoM THRs (Finnish Arthroplasty Society 2015).

ARMD is the most frequent cause of revision surgery among patients with large-diameter head MoM THR (Finnish Arthroplasty Register n.d.). Despite the implantations of MoM THRs having ceased, there are a large number of patients with MoM THR still in situ and these patients require regular follow-up. Earlier studies have shown the relationship between high whole blood (WB) metal ion levels and failure of the MoM THR (Hart et al. 2014). Therefore, WB metal ion assessments have been used to detect ARMD, first to predict the failure of the implant and second to evaluate patients’ metal ion burden (Hannemann et al. 2013, Finnish Arthroplasty Society 2014).

It is recommended that patients with large-diameter head MoM THR should be regularly monitored with clinical examination, and when necessary with metal ion measurements and MARS-MRI (magnetic artifact reduction sequence-MRI) (Medicines and Healthcare products Regulatory Agency 2012, Health Canada 2012, European Federation of National Associations of Orthopaedics and Traumatology [Effort] 2012). However, it is unclear for how long and how often (interval) patients with a large-diameter head MoM THR should be screened. There are reports of repeated metal ion measurements in a few MoM implants (van Der Straeten et al. 2013b, Reito et al. 2014), which have shown that it is useful to perform regular metal ion measurements. The tribology and failure rate of different designs of MoM THR prosthesis varies (AOANJRR 2016). Therefore, studies with different implant designs are needed. As far as we know, there are no study reports on repeated whole blood metal ion measurements after implantation of the large-diameter head ReCap-M2A-Magnum THR.

The primary aims of our study were therefore to investigate:

1. if there is a substantial change in whole blood Co or Cr levels in repeated measurements performed a mean 24 months (7 to 36) after the initial measurement in patients operated on with ReCap-M2A-Magnum THR; and
2. what proportion of patients with unilateral ReCap-M2A-Magnum THR have whole blood Co or Cr levels exceeding the safe upper limits in a mean 2-year time interval in the repeated measurements (chromium (Cr) 4.6 ppb, cobalt (Co) 4.0 ppb) (van der Straeten et al. 2013a).

## Patients and methods

We established a screening program at our institution for MoM THR to identify patients with ARMD. The screening was done according to the follow-up protocol recommended by the Finnish Arthroplasty Society (2014). The screening included an Oxford Hip Score (OHS) questionnaire, anteroposterior and lateral radiographs of the hip, and whole blood (WB) Cr and Co ion concentration measurements. Patients with moderate or poor OHS score, and/or patients with WB Cr or Co concentration > 5 ppb were referred for MRI using magnetic artifact reduction sequence (MARS). These patients were also clinically examined by a senior orthopedic surgeon at our outpatient clinic. Revision surgery for ARMD was considered if the patient had severe hip symptoms, such as pain, clicking, and swelling, and there was a clear pseudotumor on MRI. Revision surgery was also considered if an asymptomatic patient had very high WB metal ion levels (> 10 ppb) to avoid symptoms of Co poisoning (Rizzetti et al. 2009). All patients who were not revised were scheduled for annual or biennial repeat visits. Borderline cases were evaluated more frequently.

**Figure 1.**
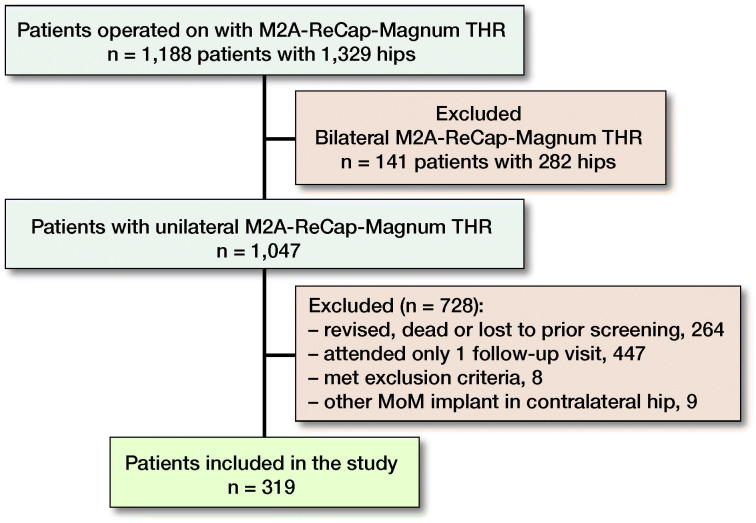
Flow chart of the study.

For this study, we identified all patients with unilateral ReCap-M2A-Magnum implants (1,047 patients). The stem used was the Biomet Bi-Metric (Biomet Orthopedics Inc, Warsaw, IN, USA). From the 264 patients without any ion measurements, 70 had been revised and 85 had died. Of these, 7 patients had been revised first and had died afterwards (Figure 1). The rest of these patients with a unilateral ReCap-M2A-Magnum implant without any ion measurements were lost to follow-up. 783 patients made the first follow-up visit including WB metal ion measurements. From the 111 patients without the second follow-up visit 12 patients had been revised, 3 had died, and 96 were lost to follow-up (Figure 1). 336 patients (336 hips) had undergone 2 follow-up visits. 9 patients had bilateral MoM hip devices and were excluded.

All participating patients had their blood samples taken from the antecubital vein using a 21-gauge BD Vacutainer Eclipse blood collection needle (Becton, Dickinson and Co, Franklin Lakes, NJ, USA). The first 10 mL tube of blood was used for analysis of standard laboratory tests such as C-reactive protein and erythrocyte sedimentation rate measurement. The second blood sample was taken in Vacuette NH trace elements tube (Greiner Bio-One GmbH, Kremsmünster, Austria) containing sodium heparin. Cobalt and chromium analyses from whole blood were performed using an accredited method with Inductively Coupled Plasma Mass Spectrometry (ICP-MS, VITA Laboratory, Helsinki, Finland in collaboration with Medical Laboratory of Bremen, Germany). The detection limit for Cr was 0.2 ppb and for Co 0.2 ppb. The intra-assay variation for WB Cr and Co was 2.2% and 2.7% and inter-assay variation was 6.7% and 7.9%, respectively.

### Statistics

319 patients met the criteria for this study with at least 2 repeated metal ion measurements. The mean time elapsing from the first metal ion assessment (initial measurement) to the second (control measurement) was 2.0 years (SD 0.5, range 0.6–3.0). All unilateral ReCap-M2A-Magnum patients operated on at our institution are considered here as the control group, whereas those patients with 2 WB ion measurements are referred to as the study group. A 2-sample t-test was used to test the difference in age, inclination angle, and femoral head diameter between the control and study groups. Sex distribution was compared using a chi-square test. Demographics were similar between the groups (Table 1).

**Table 1.** Comparison of demographic variables between the control group (= overall unilateral ReCap Magnum THR group, n = 1,047) and the study group (n = 319)

	Study group	Control group	p-value
Female patients (%)	59	55	0.2
Age (SD)	64 (9)	65 (10)	0.2
Median femoral head			
diameter (SD), mm	49 (4)	49 (4)	0.4
Mean acetabular inclination (SD)	43 (7)	43 (8)	1

The time elapsing from the index operation to the first metal ion measurement (initial) is referred to as follow-up time. Mean follow-up time between the index operation and the first metal ion measurement was 5.5 years (range 1.8 to 9.3 years). Patients were divided into follow-up time interval groups according to the time elapsing from the index operation to the first metal ion assessment. The time elapsing from the first metal ion measurement (initial) to the second measurement (control) in the same patient is referred to as the measurement interval. Thus total follow-up is defined as follow-up time plus measurement interval. The individual change in 2 consecutive metal ion measurements from the same patients was modelled using a random coefficient model. Log-transformed ion values were used in conditional models due to positively skewed distribution of ion levels. Results are expressed as geometric means for better interpretability. SUL values for WB Co were 4.0 ppb and for WB Cr 4.6 ppb as reported earlier (van der Straeten et al. 2013a). P-values lower than 0.05 in a 2-tailed test were considered statistically significant.

The change over a 2-year measurement interval was calculated and plotted as frequency distributions for both metal ions separately.

### Ethics, funding, and potential conflicts of interest

The study was based on the national recommendation for systematic screening of MoM THR patients given by the Finnish Arthroplasty Association (Finnish Arthroplasty Society 2014). It was a register study, and the patients were not directly contacted. Therefore, approval by the local ethical committee was not needed. This study was financially supported by the Competitive State Research Financing of the Expert Responsibility area of Tampere University Hospital. Outside this study, HM has received travel/accommodation expenses from DePuy Synthes. AE received research funding from Zimmer Biomet and DePuy Synthes and consultancy fees from Zimmer Biomet. AR reports personal fees from a paid lecture. PL, KTM, and TV have nothing to disclose. No benefits in any form have been received related directly or indirectly to this article.

## Results

There was a statistically significant decrease in repeated WB Co and Cr values (Table 2).

**Table 2.** Differences in WB CO and CR levels (ppb)

	Initial	Control	p-value
WB Co, n = 319			
median	1.4 (0.4–63)	1.1 (0.2–68)	
geometric mean	1.5	1.2	< 0.001
WB Cr, n = 317			
median	1.6 (0.6–13)	1.1 (0.3–19)	
geometric mean	1.7	1.2	< 0.001

Geometric mean of WB Co and Cr levels did not change in the 2-year follow-up group. However, there were only 6 measurements in the 2-year follow-up group. Geometric mean of WB Co and Cr values showed statistically significant decrease in the 3- to 6-year follow-up groups (Figure 2).

Figure 2.Geometric mean whole blood Co values (left) and Cr levels (right) divided across the follow-up time before initial measurement.

**Figure:**
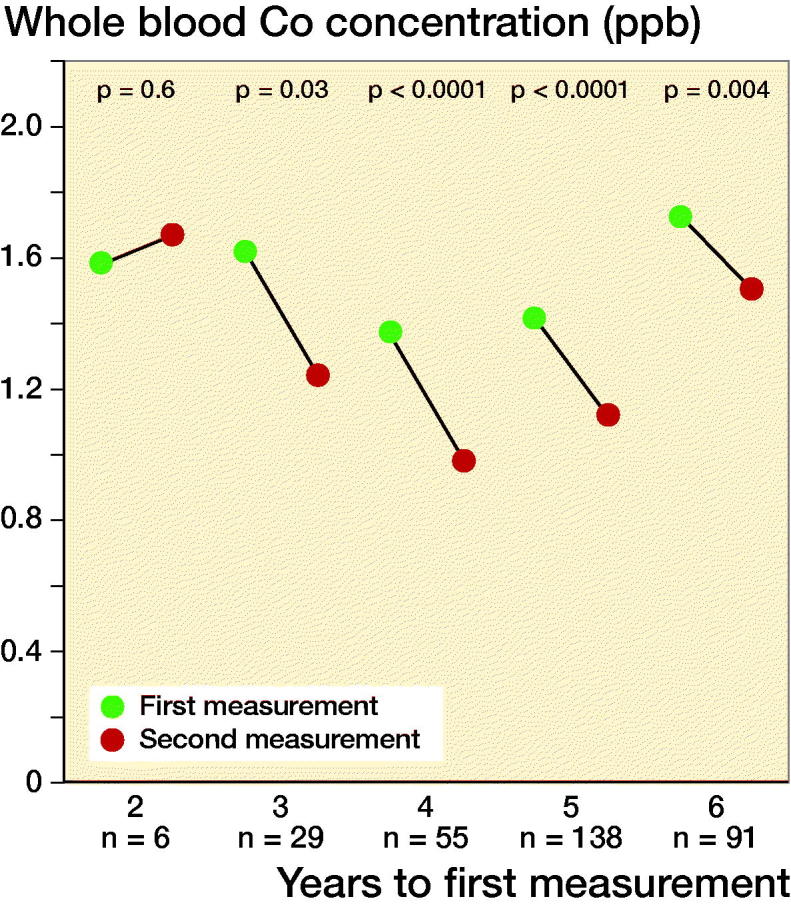

**Figure:**
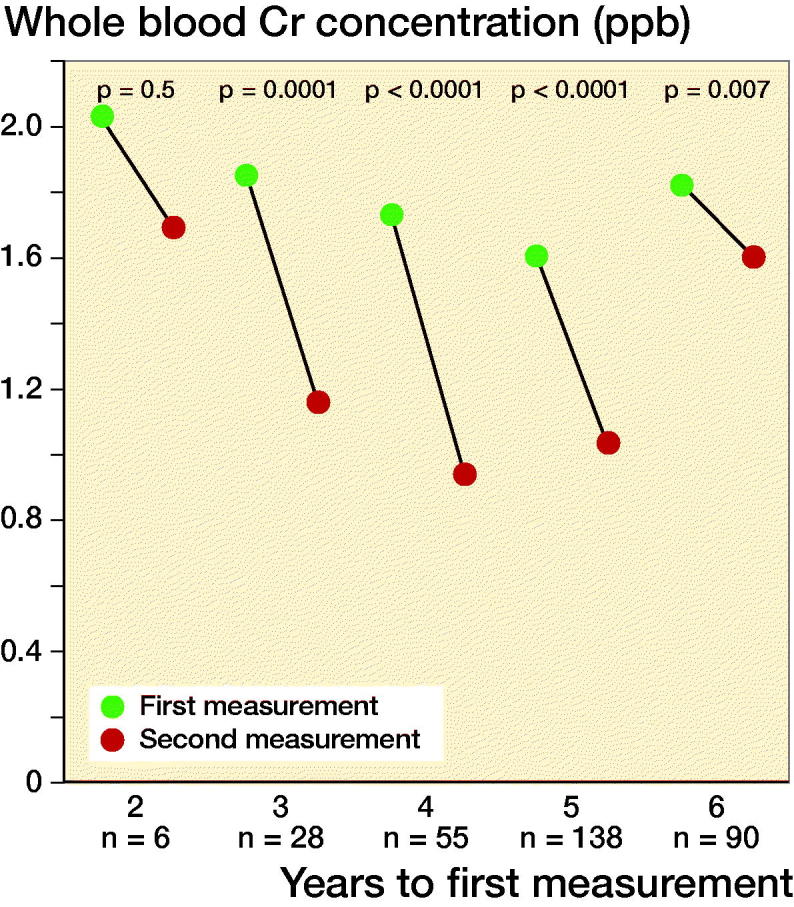

Both WB Co and Cr concentrations remained within ±1 ppb of their initial value in most patients (86% for Co, 81% for Cr), with no trends towards increasing values (Figure 3).

Figure 3.Frequency distribution of change in whole blood Co levels (left) and Cr levels (right) compared with the initial measurement.

**Figure:**
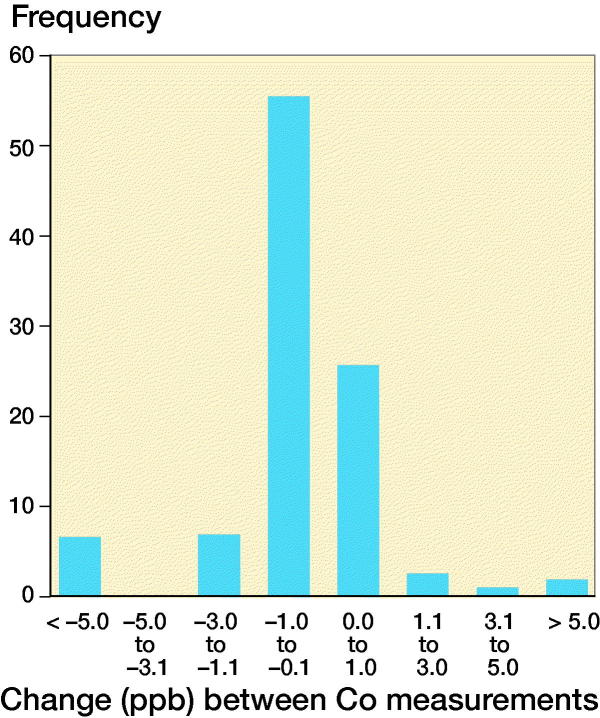

**Figure:**
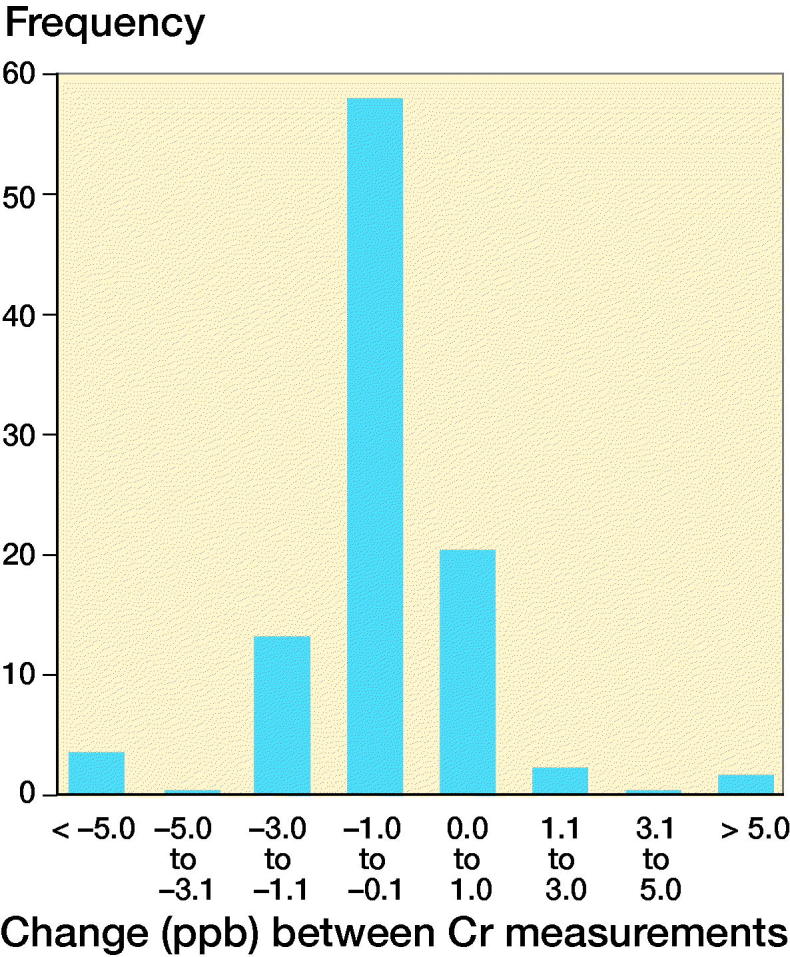

6.6% of the Co ion measurements exceeded SUL at the initial measurement. The proportion increased slightly, being 7% at the control measurement. The proportion of SUL exceeding Cr ion levels decreased during the measurement interval from 5% to 4%.

**Figure 5.**
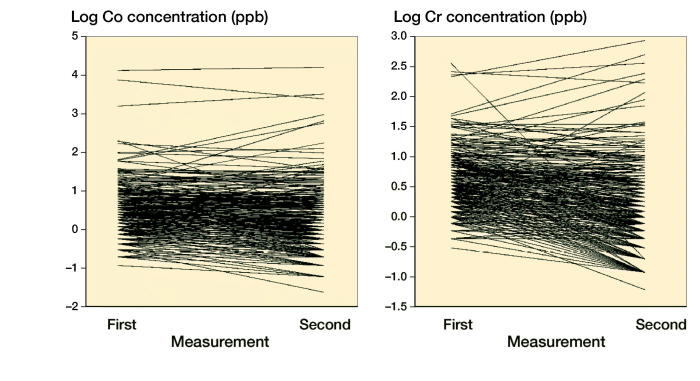
Spaghetti plots for individual Co and Cr values at initial and control measurements. Values are naturally log-transformed.

The Co and Cr levels decreased over time and stayed mostly below the SUL if the initial value was low. The exceptions were those with high values already at the start (Figure 4). The cobalt value increased from safe value to value above the safe limit in 8 patients, whereas the chromium value increased from safe value to value above the safe limit in 6 patients. Spaghetti plots for individual Co and Cr values at initial and control measurements are presented in Figure 5. Values are naturally log-transformed.

Figure 4.Changes in Co ion levels (left) and Cr ion levels (right) compared with the initial measurement.

**Figure:**
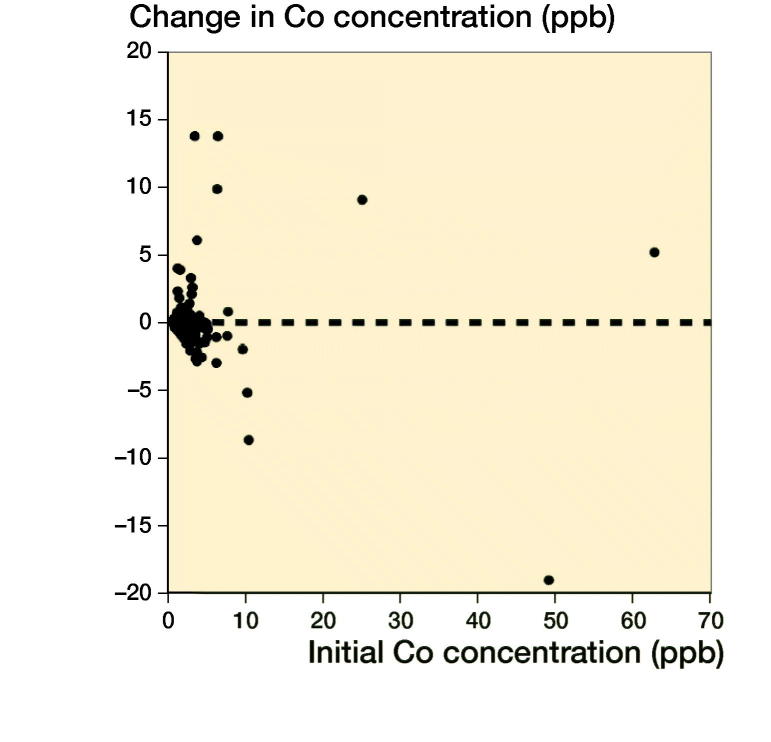

**Figure:**
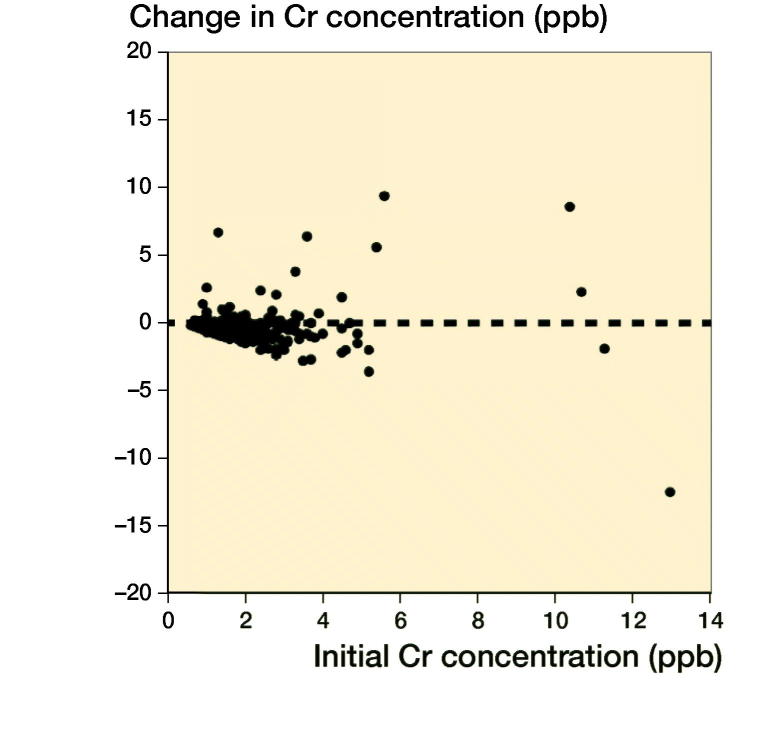

### Discussion

We found that median or geometric mean WB Co and Cr levels in repeated metal ion measurements in unilateral ReCap-M2A-Magnum patients in a mean 2-year time interval did not show notable increase. Long-term ion levels are, however, not yet known.

A limitation of our study was that the inclusion criterion used was arbitrary. We aimed to study changes in WB metal ion levels by repeated measurements, and the practical measurement interval was 2 years. The time frame from the first measurement to the second was not constant, however, in our patients. Therefore, we were compelled to select a time range, and 7 to 36 months (mean 2 years) was deemed most suitable. It is possible that a longer time range between the measurements such as 5 or 10 years might give different results. The long-term implant survivorship of the M2A-ReCap-Magnum THR, or long-term metal ion values of these patients, are not yet known. Further research is needed prior to determining whether a metal ion screening program of patients with this device should be ceased.

Another limitation of our study was also that most patients with severe hip symptoms and a clear pseudotumor in MRI had been revised and because of that did not undergo a second metal ion measurement. Most patients with very high WB ion levels initially had also been re-operated to avoid cobalt poisoning, and were not included. Therefore our study patients included mostly those with relatively low initial WB ion values. The progress of WB metal ion concentrations in patients with very high initial values is not known. Our findings are not generalizable to other MoM devices.

From the 111 patients with only 1 follow-up visit, 12 had been revised and 3 had died. We acknowledge that there were many patients with only 1 metal ion measurement. However, this is about the same proportion as in the work of Reito et al. (2016b). Some of the patients were elderly with remarkable comorbidity and they may have chosen not to come for the repeated measurements even though the possibility was provided. In Finland the healthcare system refers patients mostly to the university hospital in their own district.

The group-level results may not be relevant from a single patient perspective. For the patient, it is more relevant to know if the metal level in his/her blood is high or not, what the expected change is in a repeated measurement, and which levels will raise concern. Even if only 5% of all patients have dangerously high blood levels it may be worth measuring the blood of all patients a second time or more. Therefore, we assessed our data additionally by modelling the individual change. However, also on individual level, the increase in ion levels on repeated measurements was rare.

The Medicines and Healthcare products Regulatory Agency (MHRA) and Health Canada have recommended a cutoff level of serum cobalt and chromium of 7 ppb (MHRA 2012, Health Canada 2012). MHRA even recommends repeated testing within 3 months of abnormal results. European guidelines suggest that ion concentrations between 2 ppb and 7 ppb are of concern (EFORT 2012). The US Food and Drug Administration in the USA and Therapeutic Goods Administration in Australia do not state any cut-off ion concentration thresholds (US FDA 2013, TGA 2012). According to Hart et al. (2011) a cut-off level of 7 ppb shows good specificity, but relatively low sensitivity. Lardanchet et al. (2012) suggested a cobalt cut-off level of 8 ppb. Van der Straeten et al. (2013a) defined SUL for unilateral hip resurfacing (HR) patients at Cr 4.6 ppb and Co 4.0 ppb, and for bilateral HR patients at Cr 7.4 ppb and Co 5.0 ppb. For our study purposes we decided to use the cut-off levels suggested by van der Straeten et al. (2013a). We do not think using other cutoff levels as SUL would change our message.

Of all MoM devices, metal ion levels of ASR (DePuy, Warsaw, IN, USA) THR and HR have been scrutinized most thoroughly. Reito and co-workers (2014) assessed 254 unilateral patients, of whom 156 had received an ASR XL THR and 98 patients an ASR HR (n = 254). The second blood sample was taken 8 to 16 months after the first. In the majority of HR patients both WB Cr and Co concentrations remained within ±1 ppb in the second measurement and the majority of the values also remained below the SUL. However, in the THR group there was a significant increase in WB Co levels over the measurement interval and 32% of the patients exceeded the SUL during the measurement interval. They concluded that it is useful to perform regular WB metal ion measurements in ASR XL THR patients, although not in ASR HR patients (Reito et al. 2014). In the current study we were not able to reproduce this finding in unilateral ReCap Magnum THR patients. The measurement interval of our study was even longer (mean 2 years) than in the study of Reito et al. (2014), and the decreasing tendency of WB ion levels was clear. The poorer performance of the ASR device may explain the difference in WB ion level development compared with the ReCap Magnum THR (Seppänen et al. 2018). Our data did not include any ReCap HR devices.

In another study Reito et al. (2016b) showed that there is also a substantial increase in repeated Co and Cr level measurements in patients with bilateral ASR THR, but not with HR patients. Additionally, 21% of THR patients had WB Co ion levels already exceeding the SUL in the first measurement (Reito et al. 2016b). Our current study did not include bilateral procedures, so these previous findings could not be verified using ReCap Magnum THRs. Further research is needed concerning bilateral ReCap Magnum devices to assess WB ion level development tendency.

Matharu et al. (2015) have previously raised concern regarding variable protocols worldwide in MoM THR screening. They have suggested further research to clarify blood metal ion thresholds, and whether thresholds differ between implants (Matharu et al. 2015). Our current results strengthen this impression. Implant-specific thresholds seem to be more effective to detect ARMD (Matharu et al. 2016a, 2016b, 2017). We might need specific thresholds for different implants to have better sensitivity and specificity in ARMD screening.

Our findings imply that patients with unilateral M2A-ReCap-Magnum THR with WB metal ion levels below the SUL do not benefit from routine metal ion level screening, at least in a mean 2-year interval. We are aware that these findings cannot be generalized to other LDH MoM THR brands. Due to the previous studies of repeated metal ion measurements in ASR, the universal protocol may not be sufficient for all THR designs (Reito et al. 2016a). Implant-specific thresholds might be needed in the future to detect ARMD in different THR designs. Further, we do not know how the WB metal ion levels develop in the long term in unilateral ReCap-Magnum patients. Wear and corrosion of the bearing surface and the trunnion may well increase in long-term follow-up. Further research is needed to assess the long-term benefits of WB ion measurements and to determine the specific thresholds to detect ARMD in M2A-ReCap-Magnum THR patients. Further research is also needed to determine how frequently WB metal ion concentrations needs to be evaluated in these patients.

AR and AE designed the protocol and methods. KTM performed the surgery and recorded the intraoperative data. TV analyzed the data and did the statistics. PL, TV, and HM collected the data. PL, KTM, TV, and HM wrote the manuscript. All authors contributed to the revision of the manuscript.

*Acta* thanks Harald Brismar and Jan A N Verhaar for help with peer review of this study.
